# *IL18* Gene Variants Influence the Susceptibility to Chagas Disease

**DOI:** 10.1371/journal.pntd.0004583

**Published:** 2016-03-30

**Authors:** Daniel A Leon Rodriguez, F. David Carmona, Luis Eduardo Echeverría, Clara Isabel González, Javier Martin

**Affiliations:** 1 Instituto de Parasitología y Biomedicina López-Neyra, IPBLN-CSIC, P.T.S., Granada, Spain; 2 Clínica de Falla Cardíaca y Trasplante. Fundación Cardiovascular de Colombia, Floridablanca, Santander, Colombia; 3 Grupo de Inmunología y Epidemiología Molecular, GIEM, Facultad de Salud, Universidad Industrial de Santander, Bucaramanga, Colombia; Institute of Tropical Medicine (NEKKEN), JAPAN

## Abstract

Chagas disease is a parasitic disorder caused by the infection with the flagellated protozoan *Trypanosoma cruzi*. According to the World Health Organization, more than six million people are currently infected in endemic regions. Genetic factors have been proposed to influence predisposition to infection and development of severe clinical phenotypes like chronic Chagas cardiomyopathy (CCC). Interleukin 18 (*IL18*) encodes a proinflammatory cytokine that has been proposed to be involved in controlling *T*. *cruzi* infection. In this study, we analyzed the possible role of six *IL18* gene variants (rs5744258, rs360722, rs2043055, rs187238, rs1946518 and rs360719), which cover most of the variation within the locus, in the susceptibility to infection by *T*. *cruzi* and/or CCC. In total, 1,171 individuals from a Colombian region endemic for Chagas disease, classified as seronegative (n = 595), seropositive asymptomatic (n = 175) and CCC (n = 401), were genotyped using TaqMan probes. Significant associations with *T*. *cruzi* infection were observed when comparing seronegative and seropositive individuals for rs187238 (P = 2.18E-03, OR = 0.77), rs360719 (P = 1.49E-03, OR = 0.76), rs2043055 (P = 2.52E-03, OR = 1.29), and rs1946518 (P = 0.0162, OR = 1.22). However, dependence analyses suggested that the association was mainly driven by the polymorphism rs360719. This variant is located within the promoter region of the *IL18* gene, and it has been described that it creates a binding site for the transcription factor OCT-1 affecting IL-18 expression levels. In addition, no evidence of association was observed between any of the analyzed *IL18* gene polymorphisms and the development of CCC. In summary, our data suggest that genetic variation within the promoter region of *IL18* is directly involved in the susceptibility to infection by *T*. *cruzi*, which provides novel insight into disease pathophysiology and adds new perspectives to achieve a more effective disease control.

## Author Summary

Chagas disease is a parasitic disorder caused by the infection with the protozoan *Trypanosoma cruzi*. In Latin America, this disease represents a major public health concern, as almost 6 million people are currently infected. During the last years, great efforts have been made in health policy to control the disease; however, there is still a long way ahead to achieve this challenging goal. Most affected people remains asymptomatic after infection for the rest of their lives, but around one third of infected people may develop cardiomyopathy, a condition that reduces dramatically the quality of life and life expectancy in Chagas patients. The causes of the marked differential disease outcomes are currently unknown, but it is believed that a genetic predisposition could play a relevant role in the host. We investigated in an endemic region of Colombia whether the *IL18* gene, which is involved in the immune response to intracellular pathogens like *T*. *cruzi*, is related to a higher susceptibility to infection or disease severity. Our results suggest that *IL18* is a relevant gene in Chagas disease, and could represent a valuable insight that may help to better understand the disease pathogenesis and the development of more efficient therapeutic strategies.

## Introduction

Host genetic factors have been suggested to play an important role in the susceptibility to human infectious diseases [1]. An example of such conditions is Chagas disease, which is caused by infection of the protozoan *Trypanosoma cruzi*. Recent estimations indicate that more than 70 million people live in endemic areas for this parasite, with around 6 million people being currently infected and a reported incidence of the disease of almost 30,000 cases [2,3]. Two phases, acute and chronic, are clearly defined in Chagas disease. The early stages are characterized by acute symptoms like fever, headache or swollen lymph nodes. After 8–12 weeks from the bite, infected individuals enter the chronic phase of the disease, in which most of them will remain asymptomatic for the rest of their lives. However, around 30% of patients will develop further symptoms, including chronic cardiomyopathy and/or digestive complications [4]. During the last decade, several studies have investigated the possible role of gene polymorphisms in the predisposition to *T*. *cruzi* infection and/or chronic Chagas cardiomyopathy in patients from endemic countries, reporting promising results [5–17].

Interleukin 18 (*IL18*) is one of the genes that have been proposed to influence the development of Chagas disease. It encodes a proinflammatory cytokine that was originally described as an interferon-gamma (IFN-γ) inducing factor. Because of this, IL-18 was classified among the Th1-inducing family of cytokines, along with IL-2, IL-12 and IL-15 [18]. Due to its crucial role in the induction of IFN-γ production by T cells and NK cells, thus promoting the Th1 response, IL-18 is considered a relevant molecule for controlling intracellular pathogens [19,20].

In Chagas disease, IFN-γ is essential for parasite control during the early stages of the infection. It has been described that knockout mice for *IFNG* are highly susceptible to infection due to defective macrophage activation and nitric oxide production [21]. Interestingly, mice inoculated with *T*. *cruzi* displayed elevated IL-18 levels 6 days after infection followed by an increase of IL-12 and IFNγ [22]. Indeed, IL-18 can mediate IFN-γ induction in T cells in an IL-12 independent manner [23].

Consistent with the above, previous studies have suggested a genetic influence of both *IFNG* and *IL18* gene variants in the susceptibility to infection by *T*. *cruzi* and Chagas cardiomyopathy, respectively [9,15], adding additional evidences to the high relevance that this pathway may have in Chagas disease development.

Taking into consideration all this knowledge, we decided to perform a comprehensive analysis of the *IL18* variation, in a well-powered cohort from an endemic region of *T*. *cruzi*, in order to dissect the possible genetic association of the region with predisposition to infection by this parasite and/or the development of cardiomyopathy in Chagas patients.

## Materials and Methods

### Study subjects

A total of 1,171 Colombian individuals from an endemic region for Chagas disease (Guanentina and Comunera provinces, at the department of Santander localized between 5°26’ and 8°08’ north and 72°26’ and 74°32’ west) were included in this study (S1 Fig). The population in this region of Colombia is a homogeneous mixture, with no specific concentration of any ethnicity. All participants underwent a serological diagnosis for *T*. *cruzi* infection by means of the enzyme-linked immunosorbent assay (ELISA) and a commercial indirect hemagglutination test. According to the results of these tests, 576 individuals were classified as seropositive for *T*. *cruzi* antigens and 595 were classified as seronegative, with this latter group being used as controls. Subsequently, and based on the results of the clinical evaluation, an electrocardiogram and echocardiogram were recorded to detect any conduction alteration and/or structural cardiomyopathy. As a result, 175 seropositive individuals were classified as asymptomatic and 401 individuals were classified as having chronic Chagas cardiomyopathy. From this last group, Chagas patients were further subclassified accordingly to the severity of cardiomyopathy as follows: CII (n = 166, radiology indicative of light heart hypertrophy or minor ECG alterations), CIII (n = 200, moderate heart hypertrophy and considerable ECG alterations, mainly conduction abnormalities) and CIV (n = 35, severe cardiomegaly and marked ECG alterations, predominantly frequent and/or complex forms of ventricular arrhythmia). The mean age of participants was 45.86 years for seronegative individuals, 58.00 for asymptomatic individuals and 63.14 for chronic Chagas cardiomyopathy patients. The sex distribution for the entire group was 55% female and 45% male. None of the patients included in this study received any treatment (*i*.*e*. Benznidazole) for the infection.

### Ethics statement

The Ethics Committees from the ‘Universidad Industrial de Santander and Fundación Cardiovascular de Colombia’ approved this study (entitled “*Identificación de factores de riesgo genético para Cardiopatia Chagásica crónica*” [Identification of genetic risk factors for chronic Chagas cardiomyopathy] and approved on June 27th 2005 in the Act No. 15 of 2005) in accordance with the ethical standards laid down in the 1964 Declaration of Helsinki. Written informed consent was obtained from all subjects prior to participation.

### SNP selection and genotyping

In order to comprehensively analyze the possible role of *IL18* on the genetic susceptibility to Chagas disease, a total of six single-nucleotide polymorphisms (SNP) within the *locus* were selected for genotyping following a combined candidate gene/tagging strategy. These include: 1) two promoter variants (rs187238 and rs1946518) that have been reported to affect gene expression [24–27]; 2) one intronic variant (rs2043055) previously implicated in Chagas disease outcome in a Brazilian population [15]; 3) an additional promoter variant (rs360719) that has been described to interact with the transcription factor OCT-1 [28]; and 4) two intronic tag SNPs (rs5744258 and rs360722) covering the remaining variability of the *IL18* gene. These latter variants were selected with the software Haploview V4.2 [29] on the basis of both pairwise tagging (r^2^>0.80) and minor allele frequencies >0.1, using Colombian-Medellín (CLM) genotype data from the 1000 genomes phase III project (http://www.1000genomes.org) [30], encompassing the coding and promoter regions of *IL18* and considering the four previously selected candidate variants.

Genomic DNA was isolated following standard procedures and the genotyping was performed using TaqMan assays (Applied Biosystems, Foster City, California, USA) on a LightCycler 480 real-time PCR system (Roche Diagnostics, Basel, Switzerland).

### Statistical analysis

All statistical analyses were performed with the statistical software package Plink V1.07 (http://pngu.mgh.harvard.edu/purcell/plink) [31]. Deviance from Hardy-Weinberg equilibrium was determined at the 1% significance level in all groups of individuals. To test for possible allelic and genotypic associations, we analyzed the allelic, genotypic and haplotypic frequencies by comparing seronegative vs. seropositive individuals and asymptomatic vs. chronic Chagas cardiomyopathy individuals using the χ^2^ test and logistic regression analyses, when necessary. The Benjamini & Hochberg step-up false discovery rate (FDR) correction was used in all analyses to control for multiple testing. Permutation tests (10,000 permutations) were also performed in the haplotype analysis to estimate empirical P-values as implemented in Plink. Odds ratios (OR) and 95% confidence intervals (CI) were calculated according to the Woolf’s method. P-values lower than 0.05 were considered as statistically significant. Pairwise linkage disequilibrium (LD) (D’ and r^2^) and haplotypes were estimated using an expectation–maximization algorithm implemented in Haploview. In addition, to determine whether the haplotype model better explained the observed effects than the model considering the individual SNPs, we compared the goodness of fit of both models by a likelihood ratio test as described elsewhere [32].

The statistical power of our study (Table A in S1 Text) was estimated with the Power Calculator for Genetic Studies 2006 (CaTS) software (http://www.sph.umich.edu/csg/abecasis/CaTS/) [33].

## Results

**Fig 1:**
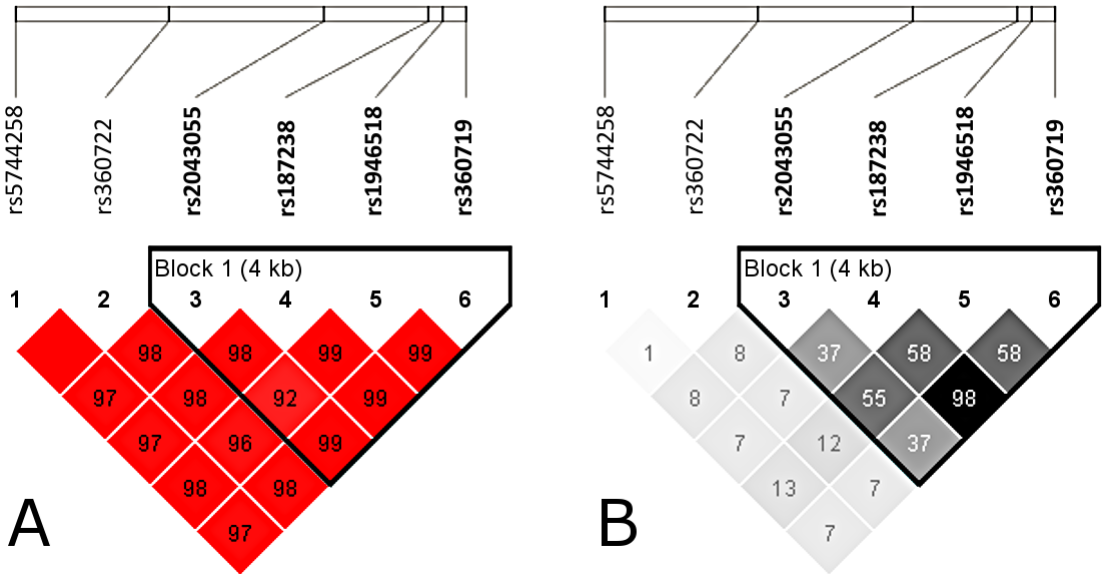
Linkage disequilibrium D’ (A) and R-Squared (B) plots estimated by using expectation-maximization algorithm in Haploview V4.2. The analysis revealed that *IL18* genetic variants were in strong LD.

The six *IL18* SNPs were in Hardy-Weinberg equilibrium in all the analyzed subgroups (P>0.01), suggesting that a possible inbreed in Guanentina and Comunera provinces is not likely. The genotyping success rate was over 95% and the allele frequencies in all cases were similar to those described for the Colombian population (CLM) of the 1000 genomes phase III project (http://www.1000genomes.org) [30]. A relatively high LD was observed throughout the gene in the analyzed population (Fig 1). Particularly, rs187238 and rs360719 showed an r^2^ value = 0.98, indicating that these two variants are almost completely linked and, consequently, they may be considered as the same marker for this study.

In order to evaluate the possible association between *IL18* gene variants and susceptibility to *T*. *cruzi* infection, the allelic and genotypic frequencies of seronegative and seropositive individuals were compared (Table 1). The allelic frequencies of four out of the six *IL18* genetic variants were significantly different between these two groups of individuals. In this regard, rs187238*C and rs360719*C were significantly increased in seronegative individuals compared to the seropositive subset (P = 2.18E-03, P_FDR_ = 5.04E-03, OR = 0.77, CI = 0.65–0.91; and P = 1.49E-03, P_FDR_ = 5.04E-03, OR = 0.76, CI = 0.64–0.90; respectively), thus suggesting that these variants are associated to protection against infection by *T*. *cruzi*. On the contrary, the frequencies of rs2043055*C and rs1946518*C were reduced in the seronegative sample set in comparison with the seropositive one (P = 2.52E-03, P_FDR_ = 5.04E-03, OR = 1.29, CI = 1.10–1.53; and P = 0.0162, P_FDR_ = 0.0243, OR1.22, CI = 1.04–1.44; respectively), indicating that they are associated with a higher infection risk. No statistical significance was observed when the allelic and genotypic frequencies of both rs5744258 and rs360722 were compared between seropositive and seronegative individuals.

**Table 1:** Genotype and allele distribution for *IL18* polymorphisms in seronegative and seropositive individuals.

			Genotype. N (%)				Allele test		
SNP	1|2	Group (N)	1|1	1|2	2|2	MAF %	P	P*	OR [95% CI]
**rs5744258**	C|G	Seronegative (592)	5 (0.84)	127 (21.45)	460 (77.70)	11.57			
		Seropositive (570)	2 (0.35)	128 (22.46)	440 (77.19)	11.58	0.9952	0.9952	1.00 [0.78–1.29]
**rs360722**	T|C	Seronegative (591)	7 (1.18)	123 (20.81)	461 (78.00)	11.59			
		Seropositive (572)	11 (1.92)	119 (20.80)	442 (77.27)	12.33	0.5850	0.7021	1.07 [0.83–1.38]
**rs2043055**	C|T	Seronegative (591)	74 (12.52)	276 (46.70)	241 (40.78)	35.87			
		Seropositive (568)	98 (17.25)	281 (49.47)	189 (33.27)	41.99	**2.52E-03**	**5.04E-03**	1.29 [1.10–1.53]
**rs187238**	C|G	Seronegative (590)	101 (17.12)	276 (46.78)	213 (36.10)	40.51			
		Seropositive (572)	67 (11.71)	259 (45.28)	246 (43.01)	34.55	**2.18E-03**	**5.04E-03**	0.77 [0.65–0.91]
**rs1946518**	C|A	Seronegative (588)	123 (20.92)	309 (52.55)	156 (26.53)	47.19			
		Seropositive (572)	157 (27.45)	283 (49.48)	132 (23.08)	52.19	**0.0162**	**0.0243**	1.22 [1.04–1.44]
**rs360719**	C|T	Seronegative (593)	101 (17.03)	280 (47.22)	212 (35.75)	40.64			
		Seropositive (572)	67 (11.71)	258 (45.10)	247 (43.18)	34.27	**1.49E-03**	**5.04E-03**	0.76 [0.64–0.90]

The marked difference of the average age between the different subgroups of patients (*i*.*e*., seronegative and seropositive individuals) could represent a limitation in this study, as Chagas disease is a parasitic disorder in which patients could develop symptoms many years after the infection [3,4]. To control for this possible confounding factor, we decided to perform a logistic regression analysis accordingly with the serological status using age as covariate. The statistical significance was maintained in this analysis thus supporting the consistency of our results (Table B in S1 Text).

Due to the high LD among the four associated SNPs, which are located either within or nearby the promoter region, dependency of the associations could be masking a possible unique causal variant. To check this, conditional logistic regression analyses were conducted by conditioning each associated SNP to the remaining variants (except for rs187238 that was almost completely dependent to rs360719). The results of these analyses pointed to rs360719 as the most likely causative variant of the *IL18* association with Chagas disease, as the statistical significance of both rs2043055 and rs1946518 were clearly lost after conditioning to it, and a trend was maintained when conditioning rs360719 on these two latter variants (Table 2).

**Table 2:** Conditional logistic regression analysis for the *IL18* polymorphisms in seronegative and seropositive individuals.

SNP	P-value	P-value add to rs360719	P-value add to rs2043055	P-value add to rs1946518
rs360719^‡^	**1.49E-03**	NA	0.0996	0.0548
rs2043055	**2.52E-03**	0.1839	NA	0.1035
rs1946518	**0.0162**	0.8918	0.6340	NA

^‡^ Same signal as rs187238

Subsequently, in order to investigate the possible association between *IL18* and chronic Chagas cardiomyopathy, we compared the allelic and genotypic frequencies of the *IL18* SNPs between seropositive asymptomatic individuals and chronic Chagas cardiomyopathy individuals (Table 3). No statistically significant differences were observed between asymptomatic individuals and chronic Chagasic cardiomyopathy patients for any of the analyzed polymorphisms. In addition, to further evaluate the possible association between *IL18* and progression of cardiomyopathy, we compared *IL18* allelic and genotypic frequencies by grouping asymptomatic + CII individuals and CIII + CIV individuals; however, similar to that observed in the previous analysis, no statistically significant differences were yielded (Table C in S1 Text).

**Table 3:** Genotype and allele distribution for *IL18* polymorphisms in asymptomatic and chronic Chagas cardiomyopathy (CCC) individuals

			Genotype. N (%)				Allele test	
SNP	1|2	Group (N)	1|1	1|2	2|2	MAF %	P	OR [95% CI]
**rs5744258**	C|G	Asymptomatic (175)	0 (0.00)	37 (21.14)	138 (78.86)	10.57		
		CCC (395)	2 (0.51)	91 (23.04)	302 (76.46)	12.03	0.4792	1.16 [0.77–1.73]
**rs360722**	T|C	Asymptomatic (174)	4 (2.30)	29 (16.67)	141 (81.03)	10.63		
		CCC (398)	7 (1.75)	90 (22.56)	301 (75.69)	13.07	0.2409	1.26 [0.85–1.88]
**rs2043055**	C|T	Asymptomatic (173)	36 (20.81)	78 (45.09)	59 (34.10)	43.35		
		CCC (395)	62 (15.70)	203 (51.39)	130 (32.91)	41.39	0.5378	0.92 [0.71–1.19]
**rs187238**	C|G	Asymptomatic (173)	25 (14.45)	74 (42.77)	74 (42.77)	35.84		
		CCC (399)	42 (10.53)	185 (46.37)	172 (43.11)	33.71	0.4862	0.91 [0.70–1.19]
**rs1946518**	A|C	Asymptomatic (175)	45 (25.71)	80 (45.71)	50 (28.57)	48.57		
		CCC (397)	87 (21.91)	203 (51.13)	107 (26.95)	47.48	0.7337	0.96 [0.74–1.23]
**rs360719**	C|T	Asymptomatic (174)	26 (14.94)	75 (43.10)	73 (41.95)	36.49		
		CCC (398)	41 (10.30)	183 (45.98)	174 (43.72)	33.29	0.2937	0.87 [0.67–1.13]

Finally, we also investigated a possible haplotype effect between the associated SNPs and susceptibility to *T*. *cruzi* infection (Table 4). Five possible haplotypes were observed (rs2043055|rs187238|rs1946518|rs360719: TCAC, CGCT, TGCT, TGAT and CGAT), with the haplotypes TCAC and CGCT showing the higher frequencies (37.30% and 37.40%, respectively). In relation to the seronegative vs seropositive analysis, the frequency of TCAC was increased in the former individuals, being this difference statistically significant (P = 1.50E-03, P_FDR_ = 7.50E-03, OR = 0.76, CI = 0.65–0.90). On the other hand, the frequency of CGCT was significantly lower in seronegative individuals compared with seropositive individuals (P = 0.0116, P_FDR_ = 0.0290, OR = 1.25, CI = 1.05–1.47), whereas the frequencies of TGCT, TGAT, and CGAT did not differed significantly between seropositive and seronegative individuals. Similar results were observed when the haplotype analysis was performed using permutation test with 10,000 permutations instead of Chi-square (Table 4). However, the haplotype model did not better explain the *IL18* association to risk of infection than the model considering the SNPs independently (likelihood P-value = 0.1454), indicating no additive effects (that is, the associated haplotypes were a consequence of the independent associations of the considered variants).

**Table 4:** *IL18* haplotype analysis of seropositive and seronegative individuals.

	Seropositive		Seronegative					
Haplotype	N	(%)	N	(%)	P	*P_FDR_	**P_PERM_	OR [95% CI]
TCAC	391	34.10	479	40.50	**1.50E-03**	**7.50E-03**	**4.90E-03**	0.76 [0.65–0.90]
CGCT	458	40.00	413	35.00	**0.0116**	**0.0290**	**0.0428**	1.25 [1.05–1.47]
TGCT	138	12.10	144	12.20	0.9222	0.9222	1.000	0.99 [0.77–1.27]
TGAT	137	12.00	134	11.30	0.6148	0.7685	0.9758	1.00 [0.89–1.12]
CGAT	18	1.60	12	1.00	0.2262	0.3770	0.6380	1.52 [0.74–3.13]

^**‡**^**Order of SNPs:** rs2043055|rs187238|rs1946518|rs360719

*P value after Benjamini & Hochberg step-up false discovery rate correction.

**Permutation test P-value for 10,000 permutations.

In relation to the haplotype analysis according to the presence/absence of chronic Chagas cardiomyopathy and/or to the progression of cardiomyopathy, no statistically significant differences among different subgroups of individuals were observed (Tables D and E in S1 Text).

## Discussion

This study evidenced that four genetic variants, namely rs2043055, rs187238, rs1946518 and rs360719, are statistically associated to differential risk of infection by *T*. *cruzi* in a Colombian population. However, our data suggested that the association is mainly driven by a single SNP, likely rs360719. Evidences supporting this fact include: 1) this *IL18* variant showed the most significant P-value and the higher effect size; 2) the statistical significance of both rs2043055 and rs1946518 was lost after conditioning on rs360719, whereas a trend towards significance was clearly observed for rs360719 after conditioning on rs2043055 or rs1946518; 3) no improvement in the goodness of fit for the model considering the association with rs360719 was observed for any of the haplotypic models; and 4) this *IL18* variant has a demonstrated functional implication in the gene expression [28].

On the other hand, *IL18* does not seem to be involved in later parasitic burden in the tissues of chronic infected patients. It should be noted that the analysis between symptomatic and asymptomatic patients was performed with lower statistical power than that between seronegative and seropositive patients (S1 Table). Hence, a possible type II error may not be rule out. Another possibility could be that additional genetic/environmental factors other than this gene may have a higher relevance for the disease progression [5–7,11–14].

In addition, a lack of association with infection by *T*. *cruzi* was observed for rs5744258 and rs360722. These two polymorphisms had the lower minor allele frequency and, therefore, their analysis could be limited in terms of statistical power. However, the power considering our study cohort was not reduced (92% to detect associations with OR = 1.5 at the 5% significance level) and the allele frequencies of the tested groups were very similar (rs5744258: 11.57% vs 11.58%, OR = 1.00; rs360722: 11.59% vs 12.33%, OR = 1.07). Analysis of larger cohorts would be required to definitively discard these *IL18* variants as susceptibility markers for Chagas disease.

A possible limitation in the inclusion methodology of our study could be that the seronegative group comprised individuals that underwent a seroconversion. However, in our opinion, it is more likely that seronegative individuals with putative spontaneous cure avoided antibody production due to a quick innate immune response by killer cells and macrophages instead. No consistent seroconversion rates have been reported in Chagas patients so far, and seroconverted individuals were reported only after treatment when there is not persistence in the infection [34–36]. None of the seronegative individuals included in our study were either reported to have Chagas disease or to have a previous therapy.

In addition, it should be noted that there is a considerable high prevalence of cardiac patients in our study cohort, which could suggest that the seropositive population is biased to the patients with Chagas cardiomyopathy. However, we would like to state that the participants were recruited after a medical visit to the endemic area. In this regard, individuals coming to the citation underwent serological analyses, and those showing seropositivity were subsequently subjected to electrocardiograms and further medical analyses in which they were classified as asymptomatic seropositive or CCC patients. In any case, this sample set has been used in previously published studies by our group [13] and we are confident about its homogeneity.

IL-18 is a cytokine which induces IFN-γ production activating several immune cells in response to intracellular pathogens, including *T*. *cruzi* [20]. IL-18 was shown to play an important role in early immunity to Chagas disease [22,23]. Moreover, the susceptibility to *T*. *cruzi* depends on the capability of releasing IFN-γ during early stages of infection and this is directly related to release of IL-18 during this phase [37]. Regarding this, it has been reported that rs360719 may be located within a repressor site of the gene, and individuals carrying the C allele showed a higher *IL18* expression due to the creation of a binding site for the transcription factor OCT-1 [28]. This is consistent with the protective role that we observed for this allele in Chagas disease development, and support the hypothesis that major IL-18 levels could increase parasite clearance in early stages of infection. Besides, our results are also in concordance with a previous study performed by our group reporting an association between *IFNG* and susceptibility to infection by *T*. *cruzi* [9]. Altogether, these findings clearly point to IL-18 along with IFN-γ as crucial players in the immune response against infection by this parasite. In any case, additional functional analyses are needed to confirm this assumption, and to have an accurate estimation of the putative IL-18 and IFN-γ levels that may discriminate the different subgroups of Chagas patients from each other and from the healthy population.

On the other hand, a previous study showed that *IL18* rs2043055 may modulate Chagas disease severity in a Brazilian population [15]. In our study we were not able to find an association of any of the analyzed *IL18* SNPs (including this one) with the severity of Chagas disease. We speculate that the discrepancy could be due to a different genetic background between the analyzed cohorts from Brazil and Colombia. Despite being both populations a mixture from Amerindian, west-European and African populations, the proportion of these ancestries could differ between them, which would affect the LD and haplotypic block architecture across the genome [38–40]. It would be interesting, therefore, to examine whether the association described for rs2043055 is dependent upon rs360719 in the Brazilian population, as our data suggest based on the LD structure observed in our Colombian cohort. In any case, both studies open a new window to understand differences in Chagas disease outcome and susceptibility. Another explanation for the observed differences between both studies could be the existence of different *T*. *cruzi* strains in the studied regions from Colombia and Brazil, as the two strains present in such areas (I and II, respectively) have been described to be implicated in CCC development and severity [41]. The analysis of the specific strains affecting both our population and the Brazilian one was out of the scope of this study, but it could represent an interesting future complementary analysis to this reported here.

The influence of *IL18* gene variants on the susceptibility to infection or severity of other protozoan infectious diseases has been also evaluated. In this context, a weak association between the *IL18* SNP rs1946519, and a higher risk to develop Leishmaniasis was described in Iranians [42]. Nevertheless, the authors did not find evidence of association between Leishmania infection and rs187238, which was associated with *T*. *cruzi* infection in our study. As stated before, the discrepancy could be due to population-specific genetic architectures within the gene, but also to the fact that our study had a considerably higher statistical power.

Additionally, the possible role of the *IL18* gene variants rs187238 and rs1946518 in severe malaria anemia and mortality were also investigated in a Kenyan children population [43]. The authors of that study observed that homozygosity for the rs1946518*A allele conferred protection against severe malaria, and that the allelic combination of rs187238*G and rs19463518*C had a higher frequency in the severe malaria group compared to the non-severe group [43]. In our study, the frequency of the AA genotype for rs1946518 was increased in asymptomatic individuals compared to chronic Chagas cardiomyopathy patients, but this difference was not statistically significant. Similarly, the frequency of the rs187238*G|rs19463518*C haplotype was increased in chronic cardiomyopathy individuals compared with asymptomatic patients, although the difference did not reach statistical significance either. Additional studies encompassing larger cohorts of seropositive patients with different degrees of disease severity may shed light into these putative associations.

*IL18* gene variants have also been evaluated in other infectious conditions such as hepatitis B or C viruses. Cumulating data indicate that IL-18 may influence the clearing of the viral load [44–46], as well as the severity of the infection in some cases of hepatic carcinomas or cirrhosis [26,47,48]. In this context, it has been proposed that differential expression levels of *IL18* could be directly involved in the predisposition to infection by the above mentioned viruses and in the severity of hepatitis [26,44–48], consistent with what we observed in Chagasic patients.

In conclusion, our results suggest that *IL18* variation plays an important role in the susceptibility to infection by *T*. *cruzi*, probably by influencing IL-18 production during the immune response in the early stages of the infection. The promoter polymorphism rs360719 is likely the causal variant of this association, at least in the Colombian population. In any case, further studies on this gene on different ancestries and larger samples sizes, as well functional analyses, would be desirable to validate our findings.

## Supporting Information

S1 Fig Location of the Colombian endemic regions analyzed in this study (Guanentina and Comunera). (TIF)

S1 Text Supporting information tables. Table A. Statistical power calculation considering different effect sizes; Table B. Logistic regression analysis of IL18 polymorphisms in seronegative and seropositive individuals including age as covariate; Table C. Genotype and allele distribution for IL18 polymorphisms in early chronic Chagas cardiomyopathy (Asymptomatic + CII) and advanced chronic Chagas cardiomyopathy (CIII+CIV) individuals; Table D. IL18 haplotype analysis of asymptomatic and chronic Chagas cardiomyopathy individuals; Table E. IL18 haplotype analysis of early chronic Chagas cardiomyopathy (Asymptomatic + CII) and advanced chronic Chagas cardiomyopathy (CIII+CIV) individuals.
(DOCX)
