# Supplementary figures and images for: IL18 Gene Variants Influence the Susceptibility to Chagas Disease
Source: PLoS Negl Trop Dis. 2016 Mar 30;10(3):e0004583. doi: 10.1371/journal.pntd.0004583 (PMC4814063; doi:10.1371/journal.pntd.0004583)

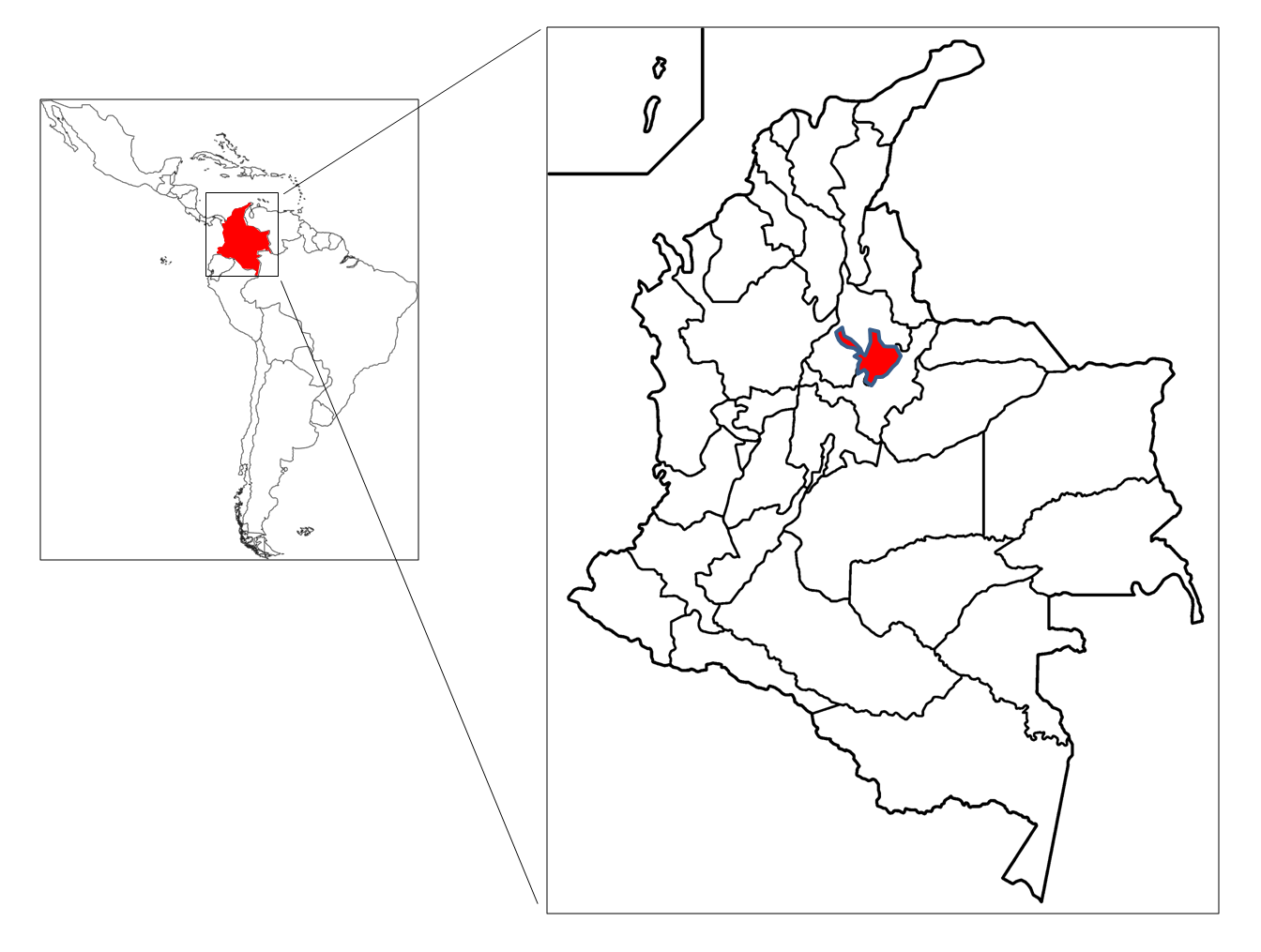

Supplement: S1 Fig — (TIF) [file pntd.0004583.s001.tif]
