# Supplementary material for: IL18 Gene Variants Influence the Susceptibility to Chagas Disease
Source: PLoS Negl Trop Dis. 2016 Mar 30;10(3):e0004583. doi: 10.1371/journal.pntd.0004583 (PMC4814063; doi:10.1371/journal.pntd.0004583)
Supplement: S1 Text — Table A. Statistical power calculation considering different effect sizes; Table B. Logistic regression analysis of IL18 polymorphisms in seronegative and seropositive individuals including age as covariate; Table C. Genotype and allele distribution for IL18 polymorphisms in early chronic Chagas cardiomyopathy (Asymptomatic + CII) and advanced chronic Chagas cardiomyopathy (CIII+CIV) individuals; Table D. IL18 haplotype analysis of asymptomatic and chronic Chagas cardiomyopathy individuals; Table E. IL18 haplotype analysis of early chronic Chagas cardiomyopathy (Asymptomatic + CII) and advanced chronic Chagas cardiomyopathy (CIII+CIV) individuals. (DOCX) [file pntd.0004583.s002.docx]

**Table A. Statistical power calculation considering different effect sizes.**

|  | **Statistical power calculation** | |
| --- | --- | --- |
|  | *T. cruzi* infection (595/576)* | Chronic Chagasic cardiomyopathy (175/401)** |
| OR=1.50 | 100% | 88% |
| OR=1.25 | 77% | 41% |
| OR=1.10 | 20% | 11% |

The estimation was performed considering a prevalence of 1.44% and a minor allele frequency of 35%.

*Analysis performed by using 595 seronegative vs. 576 seropositive individuals.

**Analysis performed by using 175 asymptomatic vs. 401 chronic Chagas cardiomyopathy individuals.

**Table B. Logistic regression analysis of *IL18* polymorphisms in seronegative and seropositive individuals including age as covariate.**

|  |  |  |  | **Allele test /Age** | | |
| --- | --- | --- | --- | --- | --- | --- |
| **SNP** | **1\|2** | **Group (N)** | **MAF %** | **P** | **P*** | **OR [95% CI]** |
| **rs5744258** | C\|G | Seronegative (592) | 11.57 |  |  |  |
|  |  | Seropositive (570) | 11.58 | 0.7581 | 0.7581 | 1.05 [0.77-1.42] |
| **rs360722** | T\|C | Seronegative (591) | 11.59 |  |  |  |
|  |  | Seropositive (572) | 12.33 | 0.6779 | 0.7581 | 1.06 [0.80-1.42] |
| **rs2043055** | C\|T | Seronegative (591) | 35.87 |  |  |  |
|  |  | Seropositive (568) | 41.99 | **6.45E-03** | **0.0129** | 1.31 [1.08-1.58] |
| **rs187238** | C\|G | Seronegative (590) | 40.51 |  |  |  |
|  |  | Seropositive (572) | 34.55 | **3.03E-03** | **9.09E-03** | 0.75 [0.62-0.91] |
| **rs1946518** | C\|A | Seronegative (588) | 47.19 |  |  |  |
|  |  | Seropositive (572) | 52.19 | **0.0220** | **0.0330** | 1.25 [1.03-1.51] |
| **rs360719** | C\|T | Seronegative (593) | 40.64 |  |  |  |
|  |  | Seropositive (572) | 34.27 | **1.88E-03** | **9.09E-03** | 0.74 [0.61-0.89] |

**Table C. Genotype and allele distribution for *IL18* polymorphisms in early chronic Chagas cardiomyopathy (Asymptomatic + CII) and advanced chronic Chagas cardiomyopathy (CIII+CIV) individuals.**

|  |  |  | **Genotype. N (%)** | | |  | **Allele test** | |
| --- | --- | --- | --- | --- | --- | --- | --- | --- |
| **SNP** | **1\|2** | **Group (N)** | **1\|1** | **1\|2** | **2\|2** | **MAF %** | **P** | **OR [95% CI]** |
| **rs5744258** | C\|G | Asy+CII (332) | 1 (0.30) | 74 (22.29) | 257 (77.41) | 11.45 |  |  |
|  |  | CIII+CIV (238) | 1 (0.42) | 54 (22.69) | 183 (76.89) | 11.76 | 0.8682 | 1.03 [0.71-1.49] |
| **rs360722** | T\|C | Asy+CII (334) | 8 (2.40) | 63 (18.86) | 263 (78.74) | 11.83 |  |  |
|  |  | CIII+CIV (238) | 3 (1.26) | 56 (23.53) | 179 (75.21) | 13.03 | 0.5432 | 1.12 [0.78-1.59] |
| **rs2043055** | C\|T | Asy+CII (331) | 59 (17.82) | 160 (48.34) | 112 (33.84) | 41.99 |  |  |
|  |  | CIII+CIV (237) | 39 (16.46) | 121 (51.05) | 77 (32.49) | 41.98 | 0.9971 | 1.00 [0.79-1.27] |
| **rs187238** | C\|G | Asy+CII (333) | 43 (12.91) | 145 (43.54) | 145 (43.54) | 34.68 |  |  |
|  |  | CIII+CIV (239) | 24 (10.04) | 114 (47.70) | 101 (42.26) | 33.89 | 0.7805 | 0.97 [0.75-1.24] |
| **rs1946518** | A\|C | Asy+CII (333) | 83 (24.92) | 153 (45.95) | 97 (29.13) | 47.90 |  |  |
|  |  | CIII+CIV (239) | 49 (20.50) | 130 (54.39) | 60 (25.10) | 47.70 | 0.9470 | 0.99 [0.78-1.26] |
| **rs360719** | C\|T | Asy+CII (332) | 43 (12.95) | 144 (43.73) | 145 (43.67) | 34.64 |  |  |
|  |  | CIII+CIV (240) | 24 (10.00) | 114 (47.50) | 102 (42.50) | 33.75 | 0.7547 | 0.96 [0.75-1.23] |

**Table D. *IL18* haplotype analysis of asymptomatic and chronic Chagas cardiomyopathy individuals.**

|  | **Asymptomatic** | | **CCC** | |  |  |
| --- | --- | --- | --- | --- | --- | --- |
| **Haplotype^‡^** | **N** | **(%)** | **N** | **(%)** | **P** | **OR [95% CI]** |
| CGCT | 143 | 40.90 | 315 | 39.50 | 0.6614 | 0.94 [0.73-1.22] |
| TCAC | 125 | 35.60 | 265 | 33.30 | 0.4534 | 0.89 [0.69-1.16] |
| TGCT | 36 | 10.20 | 102 | 12.90 | 0.2106 | 1.26 [0.84-1.88] |
| TGAT | 38 | 10.70 | 100 | 12.50 | 0.3952 | 1.00 [0.84-1.19] |
| CGAT | 6 | 1.80 | 12 | 1.40 | 0.6218 | 0.81 [0.31-2.12] |

**^‡^Order of SNPs:** rs2043055|rs187238|rs1946518|rs360719

**Table E. *IL18* haplotype analysis of early chronic Chagas cardiomyopathy (Asymptomatic + CII) and advanced chronic Chagas cardiomyopathy (CIII+CIV) individuals.**

|  | **Asy + CII** | | **CIII + CIV** | |  |  |
| --- | --- | --- | --- | --- | --- | --- |
| **Haplotype^‡^** | **N** | **(%)** | **N** | **(%)** | **P** | **OR [95% CI]** |
| CGCT | 266 | 39.90 | 191 | 40.00 | 0.9603 | 1.00 [0.79-1.27] |
| TCAC | 229 | 34.30 | 161 | 33.60 | 0.7988 | 0.97 [0.76-1.24] |
| TGCT | 80 | 11.90 | 59 | 12.30 | 0.8518 | 1.03 [0.72-1.48] |
| TGAT | 79 | 11.80 | 58 | 12.10 | 0.8828 | 1.00 [0.85-1.18] |
| CGAT | 10 | 1.50 | 8 | 1.70 | 0.7950 | 1.14 [0.46-2.84] |

**^‡^Order of SNPs:** rs2043055|rs187238|rs1946518|rs360719
